# Impact of predicted microbiota tryptophanase activity on *Cryptosporidium parvum* proliferation

**DOI:** 10.1371/journal.pone.0324042

**Published:** 2025-06-13

**Authors:** Debora Regina Romualdo da Silva, Xiaojiao Yang, Giovanni Widmer

**Affiliations:** 1 Cummings School of Veterinary Medicine at Tufts University, Department of Infectious Diseases & Global Health, North Grafton, Massachusetts, United States of America; 2 São Paulo State University (UNESP), School of Veterinary Medicine, Araçatuba, São Paulo, Brazil; 3 Diet & Chronic Diseases of Healthy Aging Research Directive, Jean Mayer USDA Human Nutrition Research Center on Aging, Tufts University, Boston, Massachusetts, United States of America; University of Illinois at Chicago, UNITED STATES OF AMERICA

## Abstract

Protozoa in the genus *Cryptosporidium* infect intestinal epithelial cells. The profile of the fecal microbiota has been shown to impact the proliferation of *Cryptosporidium parvum* in a mouse model of cryptosporidiosis and a reverse effect of the parasite on the microbiota has also been described. The mechanisms underlying this interaction are unknown. The lack of effective drugs and vaccines is motivating the search for pro- or prebiotics capable of increasing resistance to parasite proliferation in the gastrointestinal tract. To understand if and how the intestinal microbiota could be harnessed for this purpose, we tested if *C. parvum* proliferation in the mouse responds to oral administration of *Escherichia coli*. This bacterium was chosen because of its reported importance in mediating colonization resistance, because it encodes tryptophanase, an enzyme which converts tryptophan into indole, and because of the availability of an ampicillin-resistant strain expressing green fluorescent protein. Excretion of GFP^+^ *E. coli* in the feces was highly variable among mice, a phenomenon which is also observed with *C. parvum*. A positive correlation between fecal output of probiotic *E. coli* and *C. parvum* was observed. This finding may indicate that intestinal colonization with two microorganisms as different as *E. coli* and *C. parvum* responds to the same conditions in the GI tract. Consistent with an effect of the microbiota on cryptosporidiosis, the pre-infection microbiota taxonomic profile was predictive of mouse susceptibility to *C. parvum*. Contrary to the reported inhibitory effect of indole on *C. parvum*, microbiota indole production potential was positively correlated with *C. parvum* fecal output. The effect of cryptosporidiosis on the microbiota was characterized by an expansion of facultative anaerobes, particularly Gammaproteobacteria. This study is a first attempt to assess the proliferation in the mouse of a defined probiotic and quantify its effect on *C. parvum* development.

## Introduction

Cryptosporidiosis is an enteric infection of a wide range of vertebrates. The causative agents are classified in the genus *Cryptosporidium*. Human cryptosporidiosis is caused primarily by two taxonomically closely related species, *C. parvum* and *C. hominis*. Immunosuppressed mice are commonly used to model cryptosporidiosis, but immunocompetent mice infected with the rodent parasite *C. tyzzeri* have emerged as an alternative model [1,2].

The rate of *C. parvum* proliferation in experimentally infected immunosuppressed mice is highly variable between experiments, among mice originating from the same vendor, the same lot and even mice co-housed in the same cage [3]. Research into the role of the intestinal microbiota in modulating the severity of cryptosporidiosis in the mouse has uncovered extensive β diversity between mouse lots, a phenomenon previously observed by others [4]. This research also found significant associations between pre-infection fecal microbiota and the subsequent course of the infection [5]. By focusing on the fecal microbiota excreted prior to the onset of *C. parvum* oocyst shedding, it was possible to unambiguously detect an effect of the microbiota on the course of the infection. In this manner, the known effect of cryptosporidiosis on the intestinal microbiota [6–9] could be excluded.

The relevance of analyzing the effect of the intestinal microbiota on the severity of cryptosporidiosis is highlighted by the lack of effective drugs and vaccines against this potentially debilitating infection. We hypothesize that, similarly to the beneficial effect of prebiotics on the course of cryptosporidiosis in the mouse [10], selected bacterial species could increase resistance to *C. parvum* by inhibiting proliferation. Based on observations derived from metagenomic analyses of human volunteers experimentally exposed to *C. parvum* [11] and experiments in culture and in mice [12], the effect of gavaging mice with *E. coli* was measured. An ampicillin resistant *E. coli* strain expressing green fluorescent protein (GFP) was used as probiotic to enable the unambiguous quantification of the probiotic strain in the feces. This strategy also enabled us to assess the abundance of the probiotic strain relative to endogenous *Escherichia* species. Specifically, this question was examined by comparing GFP *E. coli* fecal output with the abundance of 16S sequences classified in the genus *Escherichia* or the family Enterobacteriaceae in the same fecal samples.

The goal of the experiments described here was twofold; 1) assess the feasibility of modifying the mouse gut microbiota using a simple 1-species probiotic, 2) examine the effect of this perturbation on *C. parvum* proliferation. *E. coli* was intentionally chosen as a probiotic because it encodes tryptophanase, the enzyme catalyzing the conversion of tryptophan to indole. This metabolic activity is of particular interest for understanding any role the microbiota may play in modulating *C. parvum* proliferation because indole has been reported to inhibit *C. parvum* growth in culture [12] and based on metabolomic analyses, inferred to mitigate cryptosporidiosis severity [11].

To increase the chances of *E. coli* engraftment, mice were given a 24-h course of neomycin followed by a 24-h wash-out period before *E. coli* was administered. The antibiotic treatment was not intended to clear the intestinal microbiota, but to reduce the concentration of native intestinal bacteria and favor *E. coli* engraftment.

## Materials and methods

### Mouse experiments

Twelve female CD-1 mice were randomly assigned to four groups of three mice. Starting on day −7, mice were immunosuppressed by adding dexamethasone to the drinking water at a concentration of 16 mg/l [13]. Day 0 is defined as the day of infection with *C. parvum*. Fecal pellets were collected from individual mice on day −5, −3, −2, 4, 6, 8, 10, 12, 14, 16, 19, 20 and 21, when the experiment was terminated. To perturb the intestinal microbiota and facilitate *E. coli* engraftment, mice were treated starting on day −3 for 24 h with 1 mg/ml neomycin added to the drinking water. On day −1, 48 h after neomycin was discontinued, two groups of 3 mice (group 1 and group 2) were gavaged with 6 x 10^6^ colony forming units (CFU) of ampicillin-resistant *E. coli* strain constitutively expressing GFP (strain ATCC 25922; designated here *E. coli*^*GFP*^). Mice in group 3 and group 4 received an inoculum of sterile water instead of *E. coli*. On day 0, all mice were infected by oral gavage with 2.6 x 10^4^ *C. parvum* oocysts of isolate MD [14]. Oocysts for infecting mice were purified from fecal slurries on a discontinuous gradient of Nycodenz (Alere Technologies, Oslo, Norway) as described [15].Mouse experiments were compliant with the Tufts University Animal Care and Use Committee (IACUC) protocol G2021-115. All personnel obtained IACUC clearance before entering animal facilities or working with animals. The clearance process required IACUC to evaluate the training and qualifications of personnel who intend to use live animals in research. The clearance process meets US federal and Massachusetts state law which require that all personnel is appropriately qualified to conduct work with animals. Specifically, personnel must, at a minimum: 1) understand the basic needs of each species they utilize; 2) use proper techniques when handling each species and select methods that minimize animal distress; 3) provide proper pre- and post-procedural care to animals; 4) use aseptic surgical techniques, when applicable; and 5) select and use anesthetics and tranquilizers appropriate for each species, when applicable. New personnel and supervisor are notified that they are allowed to work on the IACUC protocol. Humane endpoints were used to determine if a mouse should be euthanized; specifically, weight loss exceeding 15%, ruffled fur and hunched posture, lethargy or a combination of these symptoms. Animals were checked daily. Once a mouse presented humane endpoint criteria, it was euthanized without delay. No analgesics or anesthetics were used. One mouse in group 3 was euthanized on day 16 post-infection. The likely cause of its deteriorated appearance was cryptosporidiosis. No mouse died before meeting euthanasia criteria. In compliance with IACUC guidelines, CO_2_ inhalation was used as primary method of euthanasia followed by cervical dislocation.

### Quantification of GFP *E. coli*

Starting on day 0, one fecal pellet from group 1 (g1) and group 2 (g2) mice was homogenized in 100 μl of LB medium [16] supplemented with 100 μg/ml of ampicillin. A volume of 100 μl of this slurry was spread on LB/ampicillin plates and the plates were incubated at 37 °C for approximately 24 h. The plates were illuminated with short-wave UV light and the colonies counted (S1 Fig). To obtain accurate counts of fluorescent colonies, 2 series of 10-fold diluted fecal slurries were plated.

### Quantification of *C. parvum* DNA, *C. parvum* oocysts and bacterial DNA

Fecal DNA was extracted from individual mouse fecal pellets using the QIAamp PowerFecal Pro DNA kit from Qiagen. *C. parvum* DNA was quantified using a TaqMan quantitative PCR (qPCR) targeting the *Cryptosporidium* Oocyst Wall Protein using CryptoCOWP FWD GATGCTATCTGTCCACCAGAAT and CryptoCOWP REV CACCTGTTCCCACTCAATGTA. The probe was CryptoCOWP PRB-Cy3 5Cy3/TCTCCAGTCACAAAGGAATGCCCA/3IAbRQSp/. The QuantiNova Probe (Applied Biosystems) mastermix was used. PCR crossing points were converted into target concentration using a standard curve prepared by amplifying 19 DNA standards containing the equivalent of 10^3^, 10^4^, 10^5^ and 10^6^ oocysts. To increase the number of data point, oocyst scores estimated from acid-fast stained fecal smears [17] as described [18] were recorded on a scale ranging from 0 if no oocysts were observed to 3 when numerous oocysts per 400x microscope field were present. To combine *C. parvum* DNA concentration and microscopic oocysts scores, daily data were standardized by subtracting the mean of the values and dividing by the standard deviation. The standardized data thus represent standard deviations. The standardized scores were further normalized against sample weight.

Generic primers [19] flanking the V4 region of the prokaryotic ribosomal RNA gene were used for quantifying bacterial DNA in fecal DNA samples; forward primer 5’ GTGCCAGCMGCCGCGGTAA 3’ and reverse primer 5’ GGACTACHVGGGTWTCTAAT 3’ were used. PCR was performed with QuantiNova SYBR Green mastermix (Applied Biosystems). A standard curve was obtained by amplifying in triplicate serially diluted DNA from a synthetic bacterial population (BEI Resources, Manassas, VA, cat no. HM-782D). Total fecal DNA concentration was measured with a Qubit fluorometer (Life Technologies).

### Construction of 16S sequencing libraries

The protocol described by Kozich et al. [19] was used to amplify the V4 region of the 16S rRNA gene and tag each amplicon with a unique combination of two 8-nucleotide barcodes. The amplicon concentration was estimated using a Qubit 3 fluorometer (ThermoFischer Scientific, Waltham, Massachusetts) and the amplicons combined at approximately equal concentration. The libraries prepared in this manner were size-selected with a Pippin Prep system and sequenced paired-end 500 cycles on a MiSeq instrument operated by the Tufts Genomics core (tucf.org).

### Bioinformatics

A total of 85 16S amplicons amplified from 82 fecal DNA samples were sequenced. The total sequence yield was 5.9x10^6^. Sequence contigs were obtained from the R1 and R2 sequence pairs using *make.contigs* in mothur [20] with the trimoverlap option set to “T”. The average number of sequence contigs per amplicon was 69,711 (range 40,323−136,826). Using program *sub.sample* in mothur, amplicons were rarefied to 25,000 sequence contigs. The median length of the sequences was 249 nucleotides. Sequence de-noising was performed in mothur essentially as described [21]. Operational Taxonomic Units (OTUs) were formed with a 3% sequence dissimilarity cut-off based on the OptiClust method [22]. OTUs with an average of <1 sequence per sample were culled. Principal Coordinate Analysis plots were generated using GenAlEx [23]. The statistical significance of sample clustering was tested using ANOSIM [24] as implemented in mothur. Bonferroni correction was applied for multiple comparisons. Sequences were classified against the Silva reference taxonomy [25] (release 138.1) using *classify.seqs*. A 75% cut-off threshold was applied. To identify the bacterial taxa that differed significantly between groups, Linear Discriminant Analysis was performed using program LefSe [26] as implemented in mothur. Canonical Correspondence Analysis (CCA) and redundancy analysis (RDA) were used to assess the impact of one or multiple independent variables (predictors) on the microbiota. The choice of constrained ordination method depended on whether the dependent variables were best modeled by a linear or a unimodal model. The pseudo-F statistic [27] was calculated to test the null hypothesis of no association between the dependent and independent variables. Type I error probability were calculated using permutation. CCA and RDA were performed in CANOCO, release 5.15 [28]. Shannon diversity indices were calculated using the program *summary.single* in mothur. Program PICRUSt2 [29] was used to infer the abundance of microbiota function. Here, function refers to the Enzyme Classification system based on EC numbering. Some analyses of microbiota function refer specifically to bacterial tryptophanase (tnaA, EC 4.1.99.1). PICRUSt2 requires a BIOM-formatted input file [30] encoding OTU abundances in each sample. BIOM files were created as follows: a “shared” data matrix was generated in mothur using the *make.shared* command from a fasta file using argument label = ASV. This file was converted into a biom file using biom convert [30]. Sequence files in fastq format were deposited in NCBI’s Sequence Read Archive under BioProject number PRJNA1190088.

## Results

### Variability in *C. parvum* shedding is not unique to *C. parvum* but is also observed with *E. coli*

**Fig 1:**
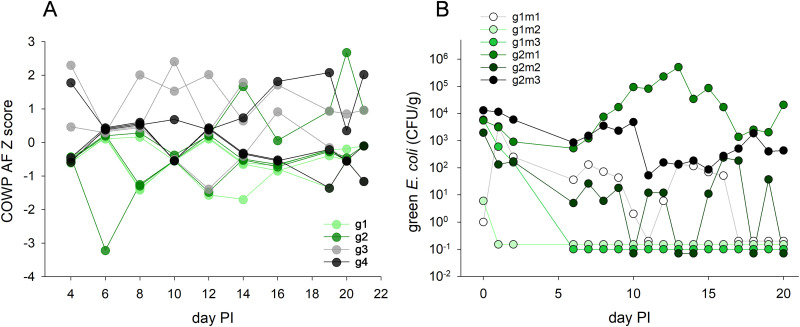
Extensive variability of *C. parvum* and *E. coli* proliferation in the mouse. **A**. Z scores of combined acid-fast counts and *C. parvum* DNA concentration estimates. Day 6, 8, 12, 14, 19 and 21 datapoints are derived from microscopy, whereas day 4, 10, 16 and 20 were obtained by qPCR. To combine both datasets, data were standardized by subtracting the mean of the values for each day and dividing the mean by the standard deviation. The Y axis scale thus represents standard deviation (Z score). g1-g4 represent four separately caged experimental groups with three mice each. Each line represents a mouse. Vertical clustering of samples, as seen in panel A, day 6, tends to occur on days for which scoring is based on microscopy. To improve visibility, overlapping datapoints were offset by 0.03 SD units. **B**. Counts of green fluorescent *E. coli* per fecal pellet vs. day post-infection for six mice in groups 1 and 2. To represent negative samples on a log scale, CFU values equal zero were replaced with 10^−1^ and offset by ± 0.05 unit to better visualize overlapping data points. Because each line represents one mouse and each point one measurement, no error bars are shown (g, group; m, mouse).

The course of *C. parvum* proliferation in the mouse can be highly variable. As previously observed [5], the infection can vary in severity from undetectable to severe, even among mice housed in the same cage. To assess whether variability is specific to *C. parvum* or is also observed with an extracellular bacterium, mice were infected with *E. coli* on day −1, i.e., 24 h prior to infection with *C. parvum* oocysts. As apparent in Fig 1, the proliferation of both microbes was highly variable between mice, even within experimental group. As can be seen in the figure, *E. coli*^GFP^ fecal concentration ranged from undetected to >10^4^, even though each mouse was infected with the same dose of bacteria and, on day 0, with the same dose of oocysts. The fact that the *E. coli* strain expressed GFP and was ampicillin resistant enabled us to quantify *E. coli*^GFP^ independently of native *E. coli* (S1 Fig) and compare fluorescent CFU counts with the relative abundance of Enterobacteriaceae sequence reads in the 16S data. This analysis showed that *E. coli*^GFP^ gavage did not increase the relative abundance of Enterobacteriaceae sequence reads. Whereas 6 of 42 group1/group2 fecal samples where Enterobacteriaceae positive, the proportion of control samples which were not given *E. coli*^GFP^ (group 3 and 4) and were positive for Enterobacteriaceae was essentially the same (7/40; Chi-square = 0.16, 1 d.f., p = 0.69). Thus, although in some mice the *E. coli*^GFP^ proliferated for the duration of the experiment (Fig 1B), *E. coli*^GFP^ did not increase the fraction of Enterobacteriaceae 16S sequences in a detectable manner.

**Fig 2:**
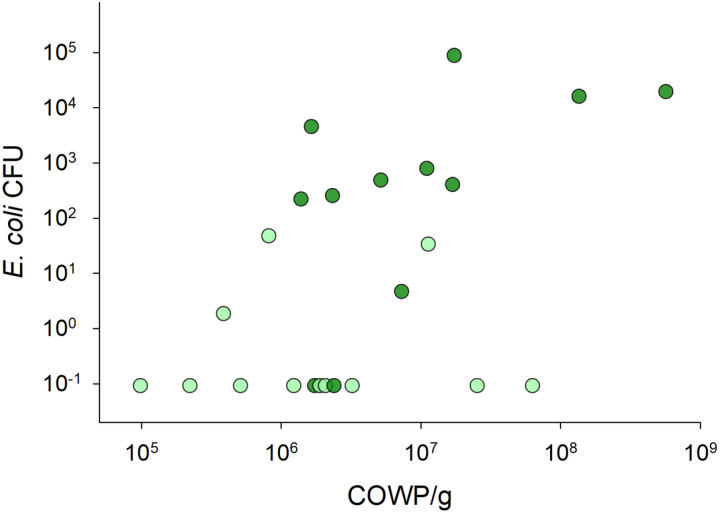
*Cryptosporidium parvum* and *E. coli* proliferation are positively correlated. Log-log plot of GFP positive *E. coli* colonies and *C. parvum* DNA concentration in feces. The analysis includes 24 samples collected between day 4 and day 20 post-infection from 6 mice pertaining to two groups of co-housed mice. The two groups were exact replicates treated in the same manner. Color indicates group; light green, group 1; dark green, group 2. Each dot represents one fecal sample. The association between the two variables is significant (Pearson r = 0.52, p = 0.01, n = 24). To avoid CFU values = 0 being excluded on the log scale, CFU counts were augmented by 0.1.

The fact that *E. coli* and *C. parvum* proliferation varied widely among mice raised the question whether the level of infection with these two organisms is correlated. GI tract conditions favoring the proliferation of both microorganisms would lead to a positive correlation, whereas mutual inhibition would result in a negative correlation. Mutual inhibition could result, for instance, by the inhibitory effect of indole against *C. parvum* as observed in culture, in mice, and inferred from fecal metabolome analyses [11,12]. The positive correlation between fluorescent CFU counts and *C. parvum* fecal DNA concentration (r = 0.52, n = 24, p = 0.01, Fig 2) is consistent with a mechanism favoring mouse susceptibility to *E. coli* and to *C. parvum*.

### Tryptophanase and *C. parvum* proliferation

Bacteria expressing tryptophanase (tnaA) are of potential interest for understanding mechanisms of interaction between the intestinal microbiota and *C. parvum*. The enzyme catalyzes the transformation of tryptophan to indole, a molecule shown to inhibit *C. parvum* in culture, in mice [12] and hypothesized to modulate susceptibility to cryptosporidiosis in humans [11]. A search of the NCBI Gene database by Boya et al. [31] found 117 bacterial species encoding this enzyme, many pertaining to the class Gammaproteobacteria. Based on the reported taxonomic distribution of tnaA, we analyzed whether *C. parvum* output was negatively correlated with the abundance of 16S sequence reads classified as Proteobacteria. Instead, as observed with the *E. coli*^GFP^ counts (Fig 2), Proteobacteria relative abundance and *C. parvum* fecal output were positively correlated (r = 0.64, n = 12, p = 0.02) (S2 Fig). Because the proportion of sequences classified as Proteobacteria, Gammaproteobacteria, Enterobacteriaceae and *Escherichia* are highly correlated, the same conclusion applies to the correlation of *C. parvum* output with either of the four taxonomic levels (Proteobacteria, Gammaproteobacteria, Enterobacteriaceae, *Escherichia*).

**Fig 3:**
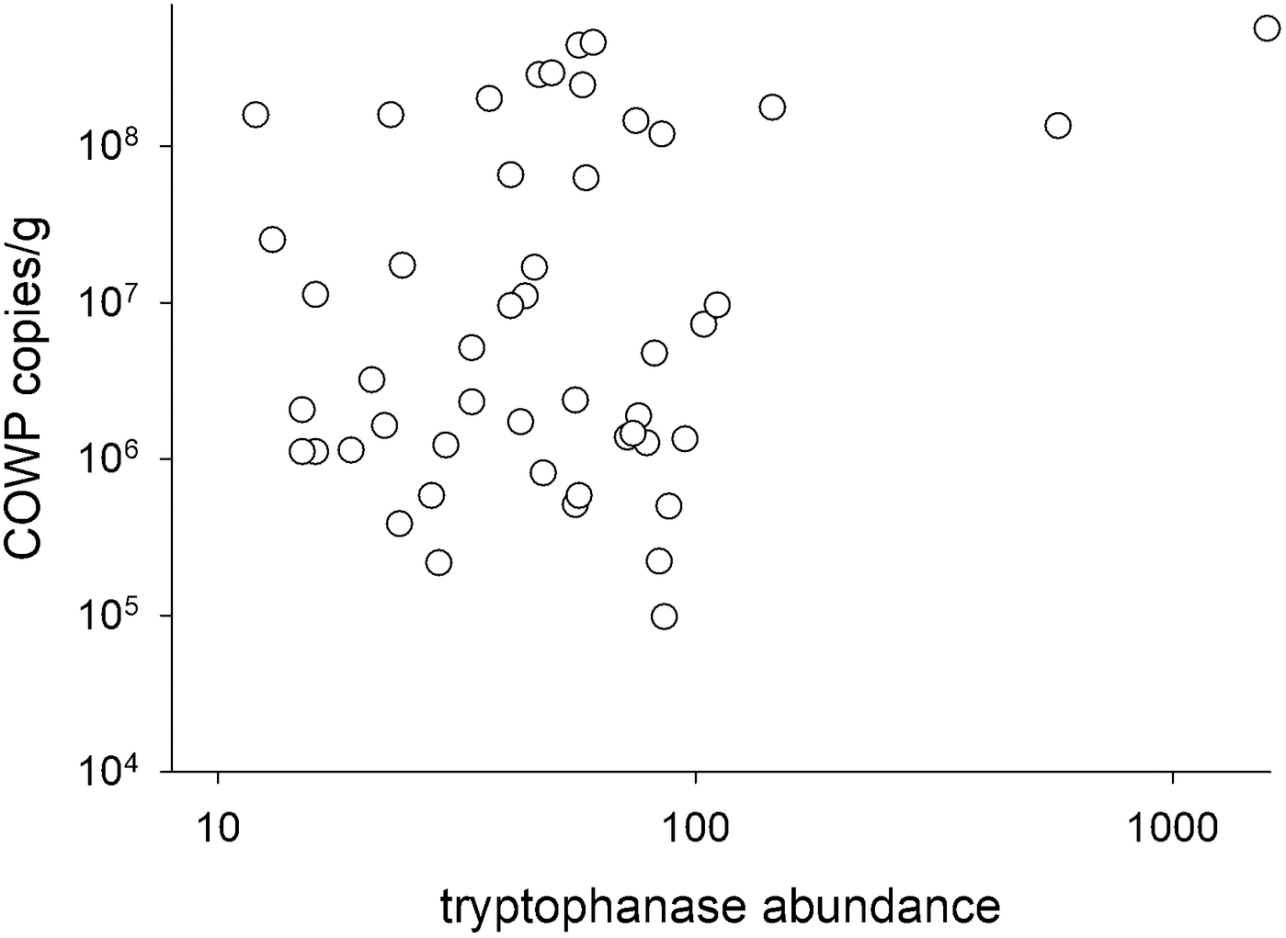
Inferred abundance of tryptophanase gene is positively correlated with *Cryptosporidium parvum* proliferation. Log-log plot shows the correlation of *C. parvum* fecal DNA and inferred microbiota tnaA abundance (Pearson r = 0.53, p = 7.9e-5, n = 48). The analysis is based on 48 samples collected from all mice between day 4 and 21 post-infection.

To further investigate a possible association between the microbiota’s predicted indole production capability and *C. parvum* proliferation, program PICRUSt2 [29] was used to infer from the 16S sequences the abundance of bacterial tnaA genes. This approach takes into consideration the entire taxonomic distribution of prokaryotic tnaA and can potentially provide a more accurate representation of the microbiota’s tryptophanase activity and indole production potential than taxonomy. Corroborating the results from the taxonomic analysis just described, a positive correlation between *C. parvum* fecal DNA and tnaA gene abundance was found in the analysis of samples for which COWP qPCR data and 16S sequence data were available (r = 0.53, p = 7.9e-5, n = 49) (Fig 3). As for the taxonomy level analyses, this result is not consistent with the view that a microbiota predicted to encode a high abundance of tnaA genes is inhibitory to *C. parvum* multiplication.

We further evaluated with RDA the association of pre-infection tnaA abundance with severity of the cryptosporidiosis. This analysis intentionally excludes post-infection samples because of the impact of the infection on the microbiota just described. According to the RDA, severity of infection only explained 6.8% of tnaA variation, equivalent to rank 195 out of 1119 enzymes included as dependent variables in the RDA (S3 Fig). In other words, 194 of 1119 enzyme functions identified by PICRUSt2 were more closely related (positively or negatively) to the severity of cryptosporidiosis. This result, together with a lack of negative correlation between inferred pre-infection tnaA abundance and *C. parvum* DNA excretion described above is consistent with the absence of a direct indole effect on *C. parvum* proliferation in the mouse.

### Two-way interaction between intestinal microbiota and severity of cryptosporidiosis

**Fig 4:**
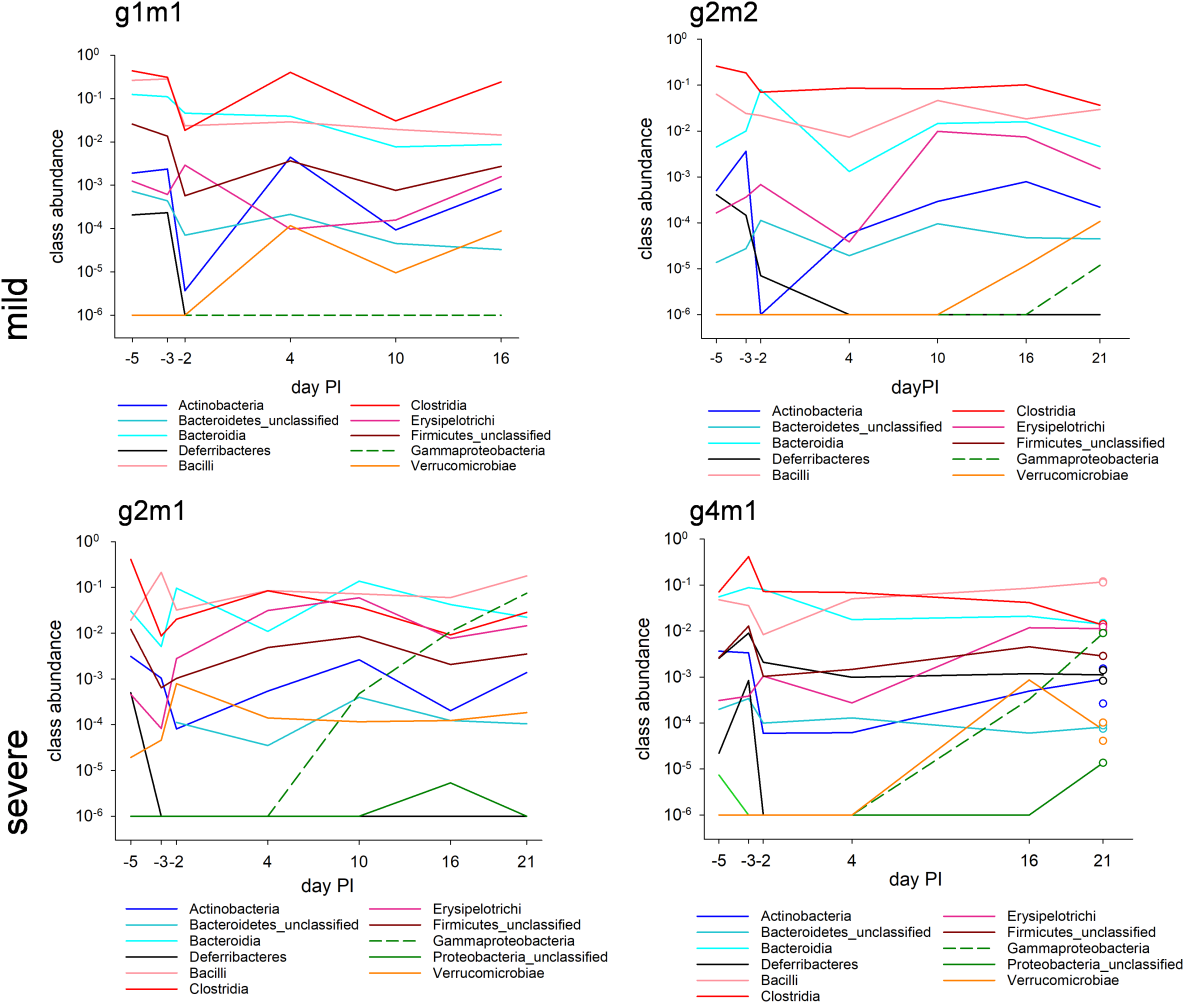
Gammaproteobacteria expansion and Clostridia depletion in mice with severe cryptosporidiosis. Class-level taxonomy of fecal microbiota in mice with mild (top) and severe (bottom) *C. parvum* infection. Taxon abundance data were adjusted for bacterial 16S gene concentration and are thus not compositional (i.e., don’t add up to 100%). Two fecal samples were collected on day 21 from mouse g4m1 and sequenced separately. The circles represent the duplicated abundance values. Their spread is thus a measure of technical variation in the data. The mice were given 1 mg/ml neomycin in the drinking water on day −3 for 24 **h.** The perturbation induced by the antibiotic treatment on day −3 and −2 is apparent. Zeros are displayed on the log scale by replacing them with 1e-6.

The positive correlation between the proportion of Proteobacteria sequences and *C. parvum* fecal output raises the possibility that a change in the intestinal microbiota resulting from the proliferation of *C. parvum* in the intestinal epithelium impacts the intestinal microbiota. This hypothesis is based on reports of the expansion of certain Proteobacteria taxa and depletion of Clostridia in response to enteric infections [32,33]. The fecal microbiota taxonomy of the two mice with the most severe cryptosporidiosis (g2m1 and g4m1) showed an increase in Gammaproteobacteria during the course of the infection (Fig 4). This change was not observed in mice which experienced a mild infection (g1m1, g2m2). LDA analysis of the 16S sequence data from days 16 and 21 post-infection, when Proteobacteria/Gammaproteobacteria sequences were most abundant, is consistent with this interpretation. Both OTUs classified as Proteobacteria-Gammaproteobacteria-Enterobacteriales which were flagged by LDA as differentially abundant between highly infected and mildly infected mice were significantly associated with the high infection group. In contrast, the taxonomy of mildly infected mice was characterized by numerous Lachnospiraceae (class Clostridia) OTUs. This family represented 127/158 OTUs with an LDA score >2 in the mildly infected group, but 0/32 OTUs in the highly infected group (S1 Table). This striking difference is also apparent in Fig 4. Following post-antibiotic microbiota recovery around day 4 post-infection, Clostridia is the most abundant class in the mildly infected mice, whereas Bacilli are more abundant in the severely infected animals. This analysis illustrates the radical change in the microbiota taxonomy associated with severe cryptosporidiosis.

**Fig 5:**
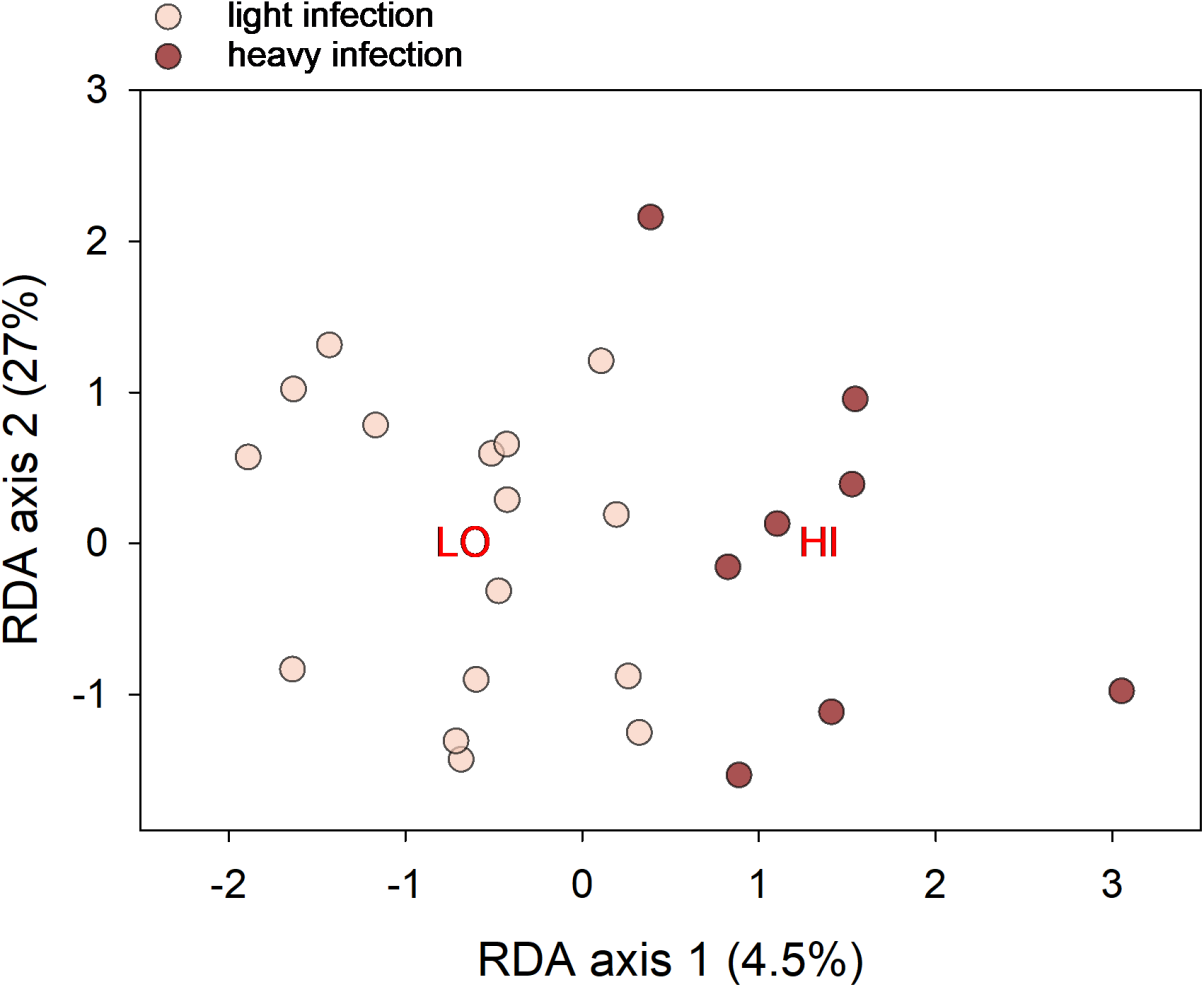
Pre-infection microbiota and severity of infection. The RDA Classified Sample biplot represents the OTU composition of 24 pre-infection fecal microbiota samples from day −5 and −3. Centroids of 8 samples from 4 mice which became severely infected (dark symbols) and from 16 samples from 8 mice which developed a mild infection (light symbols). The distance between data points represents OTU dissimilarity.

The long-term goal of developing low-cost and easily sourced pro- or prebiotics to mitigate the severity of cryptosporidiosis in humans is rooted in the hypothesis that the gut microbiota, directly or indirectly via intestinal epithelial cells, impacts *C. parvum* development. We interpret the results presented in Fig 4 as evidence of a reverse effect, of parasite multiplication on gut physiology and on the microbiota. To examine whether the opposite effect, of the microbiota on the parasite, can be detected, we analyzed the association between pre-infection microbiota and the course of the infection. A significant association would demonstrate that the microbiota indeed impacts *C. parvum* development and would strengthen previously reported observations [5]. To test for such an effect, the abundance of 733 OTUs in fecal samples collected on day −5 and −3, before the antibiotic treatment, from all 12 mice was analyzed by RDA and Linear Discriminant Analysis. For RDA, severity of infection, defined as heavy or mild based on fecal output (S2 Table), was used as the sole independent categorical variable, whereas the effect of mouse group and day of sampling were subtracted by defining them as categorical covariates. Consistent with an effect of the pre-infection microbiota, a Classified Sample plot (Fig 5) shows clustering of samples according to the subsequent course of the infection. The effect is however small as RDA axis 1 explains only 4.5% of OTU variation. Consistently, an unconstrained analysis of the same OTU table returned a statistically not significant clustering according to severity of the infection. Linear Discriminant Analysis identified 29 OTU out of 733 OTUs with a LDA score >2. These OTUs represent the taxonomic difference between pre-infection fecal microbiota of mice which developed a heavy and a mild infection, respectively. Underscoring the relatively small effect of the pre-infection microbiota on *C. parvum* development, ~ 96% (704/733) of OTUs were not associated with the extent of parasite proliferation. A Chi-Square analysis of the 29 discriminating OTUs found a significant association between taxonomic class (Bacilli vs. Clostridia vs. other/unclassified) and severity of infection (high vs. low oocyst output; Chi-Square = 9173, 2 d.f., p < 0.001).

Lastly, bacterial fecal DNA concentration estimated using generic 16S V4 primers in 44 samples for which *C. parvum* fecal output was measured was uncorrelated with the severity of cryptosporidiosis (r = 0.18, p = 0.25, n = 44). This result suggests that the abundance of bacteria in the GI tract has no impact on *C. parvum* development.

## Discussion

The original goal of the research reported here was to assess in the mouse the response of *C. parvum* infection to a simple probiotic. *E. coli* was selected as probiotic for three reasons; (a) *E. coli* has been found to be important for mediating colonization resistance in vitro and in vivo [34,35], (b) this species encodes tnaA, which catalyzes the transformation of tryptophan into indole. This molecule has been shown to inhibit parasite growth in culture and in vivo [11,12]. (c) The availability of an ampicillin-resistant *E. coli* strain expressing GFP enabled us to monitor persistence and proliferation in the mouse of the *E. coli* probiotic. The positive correlation between *E. coli*^GFP^ and *C. parvum* we report may point to a common mechanism controlling the multiplication of these microorganisms, or perhaps to a causal link, such as parasite multiplication favoring the growth of facultative anaerobes. Conversely, *E. coli* and taxonomically related bacteria could stimulate *C. parvum* development through unknown mechanisms. Regardless of the nature of the link between the growth of these microorganisms, our analyses do not show that the native microbiota and the probiotic’s indole production potential inferred from the abundance of the tryptophanase enzyme depresses parasite multiplication. Many reasons could account for this apparent discrepancy with published observations [11,12]. A primary concern is the limited resolution of 16S amplicon sequencing. Sequences classified into a family comprising tnaA-positive genera may thus not necessarily originate from a bacterium encoding this enzyme. Certain bacterial genomes may encode multiple tnaA paralogs. Also, the presence of a gene encoding this enzyme does not automatically translate into the expression of enzymatic activity and production of indole. The analysis in future experiments of the fecal metabolome, combined with a diverse set of probiotics is necessary to improve our understanding of any probiotic effect on *C. parvum* development.

An obvious limitation of the murine cryptosporidiosis model is that in the mouse, in contrast to humans, the parasite does not cause diarrhea. The Proteobacteria bloom observed in severely infected mice indicates that the mouse microbiota replicates certain features of human dysbiosis caused by enteric infection. The depletion of Clostridia and the expansion of Gammaproteobacteria is reminiscent of antibiotic-induced dysbiosis [33] and points to a loss of anaerobiosis, perhaps as a result of the damage *C. parvum* causes to the intestinal epithelium [18,36].

Similar to what we reported previously [5], the analyses described here reveal an association between the pre-infection fecal microbiota and the subsequent course of cryptosporidiosis. The proportion of pre-infection microbiota variation “explained” by the infection is however less than 5%. To some extent, this observation is not surprising since the pre-infection microbiota was severely perturbed by the neomycin treatment. Significantly, in spite of the microbiota perturbation, RDA correctly predicted which mouse eventually developed a severe or a mild infection. This outcome supports the feasibility of targeting the intestinal microbiota to reduce the susceptibility to cryptosporidiosis in at-risk populations [37,38] and in veterinary applications [39].

The ability of a healthy microbiota to protect against the colonization of the GI tract with bacterial pathogens has been explained in terms of competition for nutrients. For instance, colonization resistance has been attributed to competition for sugar molecules derived from the mucin-degrading and metabolizing activity of certain commensal bacteria [34,35]. The interaction between enteric protozoa and the intestinal microbiota remains largely unexplained. In contrast to enteric bacterial pathogens, *Cryptosporidium* parasites do not multiply in the intestinal lumen nor in the mucus layer, but only inside intestinal epithelial cells. Competition for nutrients excreted by the bacterial microbiota is thus unlikely to impact the ability of the parasite to multiply. A characteristic of *Cryptosporidium* genomes which contrasts with many prokaryotic genomes is the loss of essential metabolic functions, likely a result of metabolic parasitism. In its intracellular location, *Cryptosporidium* parasites obtain essential metabolites from the host cell, facilitated by numerous transporters encoded in their genome [40–43]. The challenge for these parasites to establish a successful infection is penetration through the mucus layer to gain entry into the enterocytes. The microbiota’s ability to metabolize mucin and modulate the thickness of the mucus layer may potentially predict the susceptibility to cryptosporidiosis. Consistent with this model, the tentative conclusion that resistance to infection with *C. parvum* was not completely abrogated by neomycin is consistent with the view that resistance to cryptosporidiosis is not a direct effect of the microbiota’s metabolic activity. Two possible mechanisms worthy of further investigation are the effect of bacterial metabolites on the intestinal epithelial cells and the modulation of mucin build-up and degradation resulting from the secretory activity of Goblet cells and bacterial mucin degradation, respectively. This working hypothesis leads us to predict that microbiota-mediated resistance to bacterial and protozoal pathogens are two different phenomena, mediated by different mechanisms of microbiota-pathogen interaction.

## Supporting information

S1 Table Linear discriminant analysis of fecal microbiota on day 16 and 21 post-infection. The fecal microbiota of severely and lightly infected mice is clearly distinct. OTUs classified in the family Lachnospiraceae are highly abundant in the latter group, whereas Enterobacteriaceae are only observed in feces of severely infected mice.
(XLSX)

S2 Table Classification of mouse samples according to severity of infection. This classification was used in the RDA described in the text.
(XLSX)

S1 Fig *E. coli*^GFP^ colonies on LB ampicillin plates illuminated with UV light. *E. coli*^*GFP*^ colonies on LB-ampicillin plates illuminated with UV light. Feces from group 1 (g1) and group 2 (g2) mice excreted on day 2 post-infection with *C. parvum* (day 3 post *E. coli*^GFP^ inoculation) were spread on LB plates. One fecal pellet was homogenized in 100 μl of LB medium [16] supplemented with 100 μg/ml ampicillin and the slurry plated on LB/ampicillin plates. The plates were incubated at 37 °C for approximately 24 h. GFP colonies were viewed with a short-wave UV transilluminator.
(JPG)

S2 Fig *Cryptosporidium parvum* fecal output is positively correlated with the relative abundance of Proteobacteria. For each of the 12 mice included in the experiment, the X axis displays mean *C. parvum* output calculated as described in Materials and Methods. The Y axis shows the mean relative abundance of Proteobacteria sequence reads averaged over 5–7 datapoints per mouse. The values of three samples with zero Proteobacteria sequence reads was set equal 10e-8. Each dot represents a mouse, colored according to the experimental group as indicated. Because most Proteobacteria sequences originate from the genus *Escherichia* (*r* = 0.96, p = 1.4e-45, n = 85), a plot of log(Escherichia) vs *C. parvum* output is essentially identical.
(JPG)

S3 Fig Pre-infection tryptophanase gene abundance does not correlate with the severity of cryptosporidiosis. The Y axis shows the percent fit explained by RDA. Each dot represents an enzyme function as predicted from 16S sequences by PICRUSt2; tnaA is highlighted in red. The analysis is based on 24 pre-infection samples collected on days −5 and −3 and 1119 predicted enzyme abundance values.
(JPG)

S1 File Raw numerical data. Used to draw all graphs and supplemental numerical figures.
(XLSX)

S2 File Raw numerical data. (DB)

## References

[1] Vinayak S, Pawlowic MC, Sateriale A, Brooks CF, Studstill CJ, Bar-Peled Y, et al. Genetic modification of the diarrhoeal pathogen Cryptosporidium parvum. Nature. 2015;523(7561):477–80. doi: 10.1038/nature14651 26176919 PMC4640681

[2] Sateriale A, Šlapeta J, Baptista R, Engiles JB, Gullicksrud JA, Herbert GT, et al. A genetically tractable, natural mouse model of cryptosporidiosis offers insights into host protective immunity. Cell Host Microbe. 2019;26(1):135-146.e5. doi: 10.1016/j.chom.2019.05.006 31231045 PMC6617386

[3] Creasey HN, Zhang W, Widmer G. Effect of caging on Cryptosporidium parvum proliferation in mice. Microorganisms. 2022;10(6):1242. doi: 10.3390/microorganisms10061242 35744762 PMC9230662

[4] Ericsson AC, Davis JW, Spollen W, Bivens N, Givan S, Hagan CE, et al. Effects of vendor and genetic background on the composition of the fecal microbiota of inbred mice. PLoS One. 2015;10(2):e0116704. doi: 10.1371/journal.pone.0116704 25675094 PMC4326421

[5] Widmer G, Creasey HN. Fecal microbiota impacts development of Cryptosporidium parvum in the mouse. Sci Rep. 2024;14(1):5498. doi: 10.1038/s41598-024-56184-1 38448682 PMC10917813

[6] Ras R, Huynh K, Desoky E, Badawy A, Widmer G. Perturbation of the intestinal microbiota of mice infected with Cryptosporidium parvum. Int J Parasitol. 2015;45(8):567–73. doi: 10.1016/j.ijpara.2015.03.005 25913477

[7] Charania R, Wade BE, McNair NN, Mead JR. Changes in the microbiome of cryptosporidium-infected mice correlate to differences in susceptibility and infection levels. Microorganisms. 2020;8(6):879. doi: 10.3390/microorganisms8060879 32532051 PMC7356575

[8] Karpe AV, Hutton ML, Mileto SJ, James ML, Evans C, Shah RM, et al. Cryptosporidiosis modulates the gut microbiome and metabolism in a murine infection model. Metabolites. 2021;11(6):380. doi: 10.3390/metabo11060380 34208228 PMC8230837

[9] Mammeri M, Chevillot A, Thomas M, Julien C, Auclair E, Pollet T, et al. Cryptosporidium parvum-Infected neonatal mice show gut microbiota remodelling using high-throughput sequencing analysis: preliminary results. Acta Parasitol. 2019;64(2):268–75. doi: 10.2478/s11686-019-00044-w 30915719

[10] Oliveira BCM, Bresciani KDS, Widmer G. Deprivation of dietary fiber enhances susceptibility of mice to cryptosporidiosis. PLoS Negl Trop Dis. 2019;13(9):e0007411. doi: 10.1371/journal.pntd.0007411 31560681 PMC6785118

[11] Chappell CL, Darkoh C, Shimmin L, Farhana N, Kim D-K, Okhuysen PC, et al. Fecal Indole as a Biomarker of susceptibility to Cryptosporidium infection. Infect Immun. 2016;84(8):2299–306. doi: 10.1128/IAI.00336-16 27245413 PMC4962629

[12] Funkhouser-Jones LJ, Xu R, Wilke G, Fu Y, Schriefer LA, Makimaa H, et al. Microbiota-produced indole metabolites disrupt mitochondrial function and inhibit Cryptosporidium parvum growth. Cell Rep. 2023;42(7):112680. doi: 10.1016/j.celrep.2023.112680 37384526 PMC10530208

[13] Yang S, Healey MC. The immunosuppressive effects of dexamethasone administered in drinking water to C57BL/6N mice infected with Cryptosporidium parvum. J Parasitol. 1993;79(4):626–30. 8331488

[14] Okhuysen PC, Rich SM, Chappell CL, Grimes KA, Widmer G, Feng X, et al. Infectivity of a Cryptosporidium parvum isolate of cervine origin for healthy adults and interferon-gamma knockout mice. J Infect Dis. 2002;185(9):1320–5. doi: 10.1086/340132 12001050

[15] Widmer G, Feng X, Tanriverdi S. Genotyping of Cryptosporidium parvum with microsatellite markers. Methods Mol Biol. 2004;268:177–87. doi: 10.1385/1-59259-766-1:177 15156029

[16] Sambrook J, Russell D. Molecular cloning: a laboratory manual. 3 ed. Cold Spring Harbor, New York: Cold Spring Harbor Laboratory Press; 2001.

[17] Ma P, Soave R. Three-step stool examination for cryptosporidiosis in 10 homosexual men with protracted watery diarrhea. J Infect Dis. 1983;147(5):824–8. doi: 10.1093/infdis/147.5.824 6842020

[18] Tzipori S, Rand W, Griffiths J, Widmer G, Crabb J. Evaluation of an animal model system for cryptosporidiosis: therapeutic efficacy of paromomycin and hyperimmune bovine colostrum-immunoglobulin. Clin Diagn Lab Immunol. 1994;1(4):450–63. doi: 10.1128/cdli.1.4.450-463.1994 8556484 PMC368287

[19] Kozich JJ, Westcott SL, Baxter NT, Highlander SK, Schloss PD. Development of a dual-index sequencing strategy and curation pipeline for analyzing amplicon sequence data on the MiSeq Illumina sequencing platform. Appl Environ Microbiol. 2013;79(17):5112–20. doi: 10.1128/AEM.01043-13 23793624 PMC3753973

[20] Schloss PD, Westcott SL, Ryabin T, Hall JR, Hartmann M, Hollister EB, et al. Introducing mothur: open-source, platform-independent, community-supported software for describing and comparing microbial communities. Appl Environ Microbiol. 2009;75(23):7537–41. doi: 10.1128/AEM.01541-09 19801464 PMC2786419

[21] Ferrari ED, Oliveira BCM, Creasey HN, Romualdo da Silva DR, Nakamura AA, Bresciani KDS, et al. The impact of physical effort on the gut microbiota of long-distance fliers. Microorganisms. 2023;11(7):1766. doi: 10.3390/microorganisms11071766 37512938 PMC10386721

[22] Westcott SL, Schloss PD. OptiClust, an improved method for assigning amplicon-based sequence data to operational taxonomic units. mSphere. 2017;2(2):e00073-17. doi: 10.1128/mSphereDirect.00073-17 28289728 PMC5343174

[23] Peakall R, Smouse PE. GenAlEx 6.5: genetic analysis in Excel. Population genetic software for teaching and research--an update. Bioinformatics. 2012;28(19):2537–9. doi: 10.1093/bioinformatics/bts460 22820204 PMC3463245

[24] Clarke KR. Non‐parametric multivariate analyses of changes in community structure. Australian Journal of Ecology. 1993;18(1):117–43. doi: 10.1111/j.1442-9993.1993.tb00438.x

[25] Quast C, Pruesse E, Yilmaz P, Gerken J, Schweer T, Yarza P, et al. The SILVA ribosomal RNA gene database project: improved data processing and web-based tools. Nucleic Acids Res. 2013;41(Database issue):D590-6. doi: 10.1093/nar/gks1219 23193283 PMC3531112

[26] Segata N, Izard J, Waldron L, Gevers D, Miropolsky L, Garrett WS, et al. Metagenomic biomarker discovery and explanation. Genome Biol. 2011;12(6):R60. doi: 10.1186/gb-2011-12-6-r60 21702898 PMC3218848

[27] Lepš J, Šmilauer P. Multivariate analysis of ecological data using CANOCO. Cambridge University Press; 2003.

[28] Braak CT, Šmilauer P. CANOCO reference manual and CanoDraw for Windows user’s guide: software for canonical community ordination. Ithaca, New York: Microcomputer Power; 2002.

[29] Douglas GM, Maffei VJ, Zaneveld JR, Yurgel SN, Brown JR, Taylor CM, et al. PICRUSt2 for prediction of metagenome functions. Nat Biotechnol. 2020;38(6):685–8. doi: 10.1038/s41587-020-0548-6 32483366 PMC7365738

[30] McDonald D, Clemente JC, Kuczynski J, Rideout JR, Stombaugh J, Wendel D, et al. The Biological Observation Matrix (BIOM) format or: how I learned to stop worrying and love the ome-ome. Gigascience. 2012;1(1):7. doi: 10.1186/2047-217X-1-7 23587224 PMC3626512

[31] Boya BR, Kumar P, Lee J-H, Lee J. Diversity of the tryptophanase gene and its evolutionary implications in living organisms. Microorganisms. 2021;9(10):2156. doi: 10.3390/microorganisms9102156 34683477 PMC8537960

[32] Litvak Y, Byndloss MX, Tsolis RM, Bäumler AJ. Dysbiotic proteobacteria expansion: a microbial signature of epithelial dysfunction. Curr Opin Microbiol. 2017;39:1–6. doi: 10.1016/j.mib.2017.07.003 28783509

[33] Rivera-Chávez F, Zhang LF, Faber F, Lopez CA, Byndloss MX, Olsan EE, et al. Depletion of butyrate-producing clostridia from the gut microbiota drives an aerobic luminal expansion of salmonella. Cell Host Microbe. 2016;19(4):443–54. doi: 10.1016/j.chom.2016.03.004 27078066 PMC4832419

[34] Spragge F, Bakkeren E, Jahn MT, B N Araujo E, Pearson CF, Wang X, et al. Microbiome diversity protects against pathogens by nutrient blocking. Science. 2023;382(6676):eadj3502. doi: 10.1126/science.adj3502 38096285 PMC7616675

[35] Pereira FC, Wasmund K, Cobankovic I, Jehmlich N, Herbold CW, Lee KS, et al. Rational design of a microbial consortium of mucosal sugar utilizers reduces Clostridiodes difficile colonization. Nat Commun. 2020;11(1):5104. doi: 10.1038/s41467-020-18928-1 33037214 PMC7547075

[36] Clark DP, Sears CL. The pathogenesis of cryptosporidiosis. Parasitol Today. 1996;12(6):221–5. doi: 10.1016/0169-4758(96)10018-1 15275201

[37] Kotloff KL, Nataro JP, Blackwelder WC, Nasrin D, Farag TH, Panchalingam S, et al. Burden and aetiology of diarrhoeal disease in infants and young children in developing countries (the Global Enteric Multicenter Study, GEMS): a prospective, case-control study. Lancet. 2013;382(9888):209–22. doi: 10.1016/S0140-6736(13)60844-2 23680352

[38] Tumwine JK, Kekitiinwa A, Nabukeera N, Akiyoshi DE, Rich SM, Widmer G, et al. Cryptosporidium parvum in children with diarrhea in Mulago Hospital, Kampala, Uganda. Am J Trop Med Hyg. 2003;68(6):710–5. 12887032

[39] Sanford SE, Josephson GK. Bovine cryptosporidiosis: clinical and pathological findings in forty-two scouring neonatal calves. Can Vet J. 1982;23(12):343–7. 17422204 PMC1790278

[40] Baptista RP, Li Y, Sateriale A, Sanders MJ, Brooks KL, Tracey A, et al. Long-read assembly and comparative evidence-based reanalysis of Cryptosporidium genome sequences reveal expanded transporter repertoire and duplication of entire chromosome ends including subtelomeric regions. Genome Res. 2022;32(1):203–13. doi: 10.1101/gr.275325.121 34764149 PMC8744675

[41] Widmer G, Lee Y, Hunt P, Martinelli A, Tolkoff M, Bodi K. Comparative genome analysis of two Cryptosporidium parvum isolates with different host range. Infect Genet Evol. 2012;12(6):1213–21. doi: 10.1016/j.meegid.2012.03.027 22522000 PMC3372781

[42] Xu R, Beatty WL, Greigert V, Witola WH, Sibley LD. Multiple pathways for glucose phosphate transport and utilization support growth of Cryptosporidium parvum. Nat Commun. 2024;15(1):380. doi: 10.1038/s41467-024-44696-3 38191884 PMC10774378

[43] Xu Z, Guo Y, Roellig DM, Feng Y, Xiao L. Comparative analysis reveals conservation in genome organization among intestinal Cryptosporidium species and sequence divergence in potential secreted pathogenesis determinants among major human-infecting species. BMC Genomics. 2019;20(1):406. doi: 10.1186/s12864-019-5788-9 31117941 PMC6532270

