# Supplementary figures and images for: Impact of predicted microbiota tryptophanase activity on Cryptosporidium parvum proliferation
Source: PLoS One. 2025 Jun 13;20(6):e0324042. doi: 10.1371/journal.pone.0324042 (PMC12165429; doi:10.1371/journal.pone.0324042)

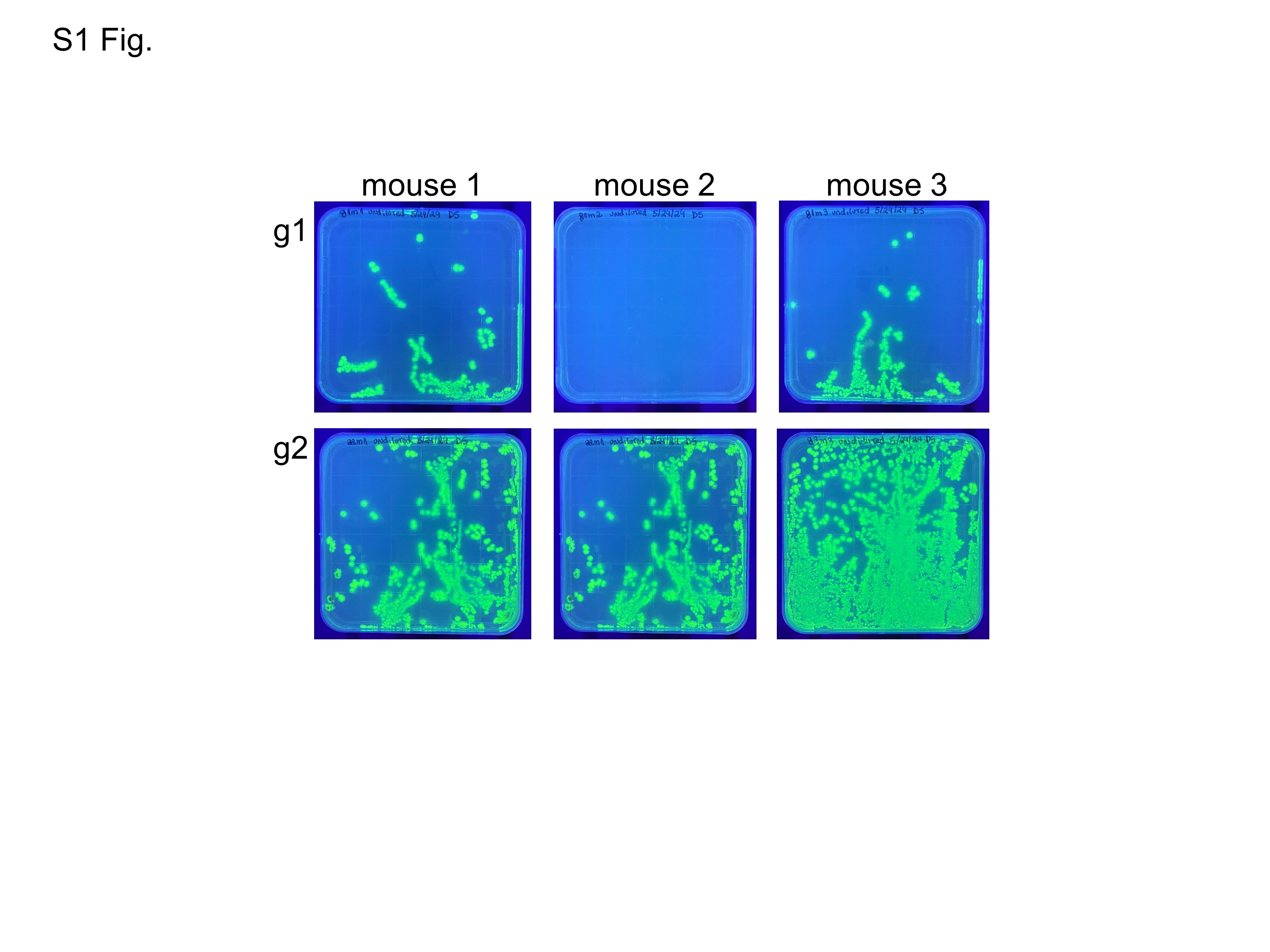

Supplement: S1 Fig — E. coliGFP colonies on LB-ampicillin plates illuminated with UV light. Feces from group 1 (g1) and group 2 (g2) mice excreted on day 2 post-infection with C. parvum (day 3 post E. coliGFP inoculation) were spread on LB plates. One fecal pellet was homogenized in 100 μl of LB medium [16] supplemented with 100 μg/ml ampicillin and the slurry plated on LB/ampicillin plates. The plates were incubated at 37 °C for approximately 24 h. GFP colonies were viewed with a short-wave UV transilluminator. (JPG) [file pone.0324042.s003.jpg]

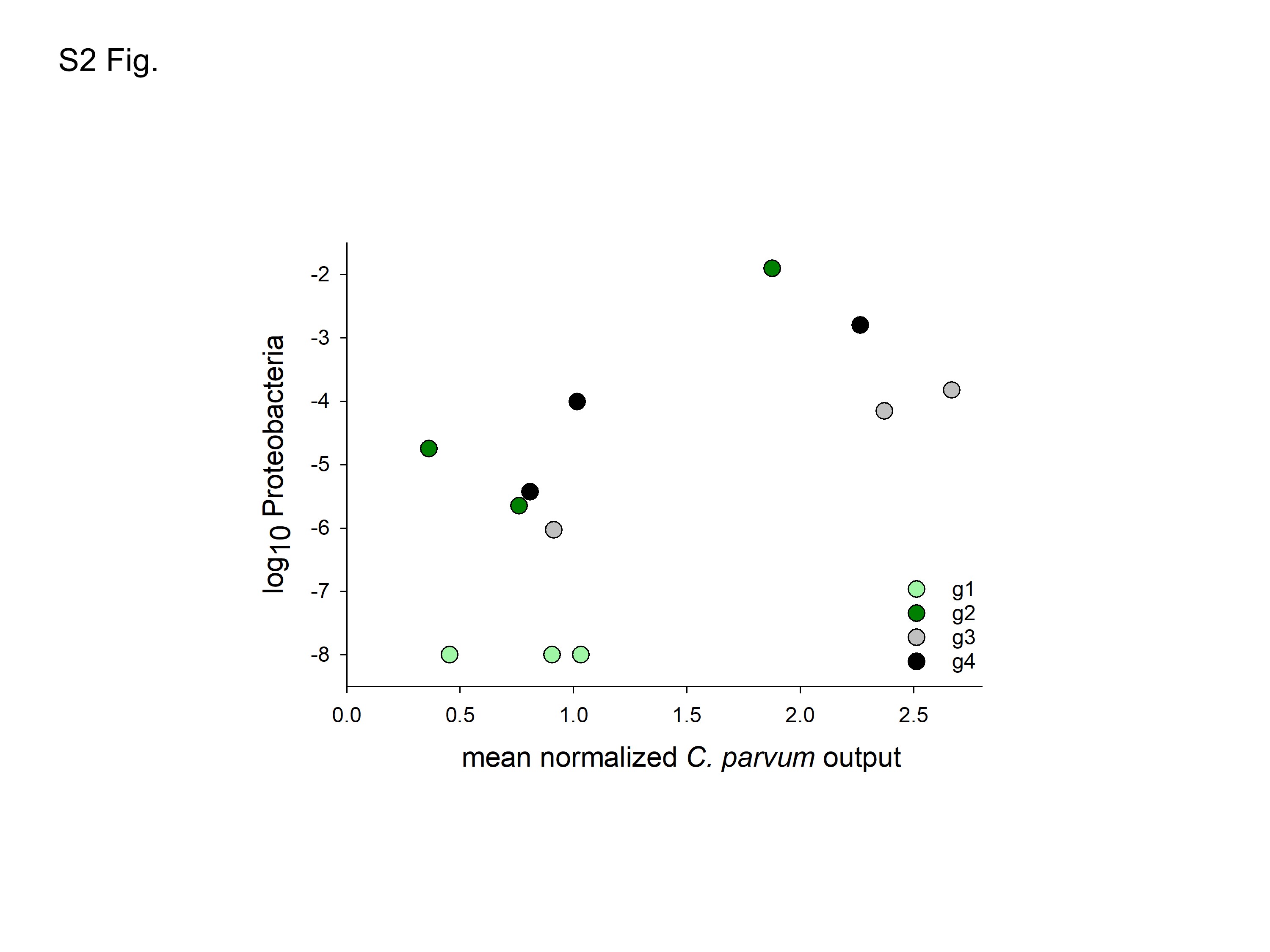

Supplement: S2 Fig — For each of the 12 mice included in the experiment, the X axis displays mean C. parvum output calculated as described in Materials and Methods. The Y axis shows the mean relative abundance of Proteobacteria sequence reads averaged over 5–7 datapoints per mouse. The values of three samples with zero Proteobacteria sequence reads was set equal 10e-8. Each dot represents a mouse, colored according to the experimental group as indicated. Because most Proteobacteria sequences originate from the genus Escherichia (r = 0.96, p = 1.4e-45, n = 85), a plot of log(Escherichia) vs C. parvum output is essentially identical. (JPG) [file pone.0324042.s004.jpg]

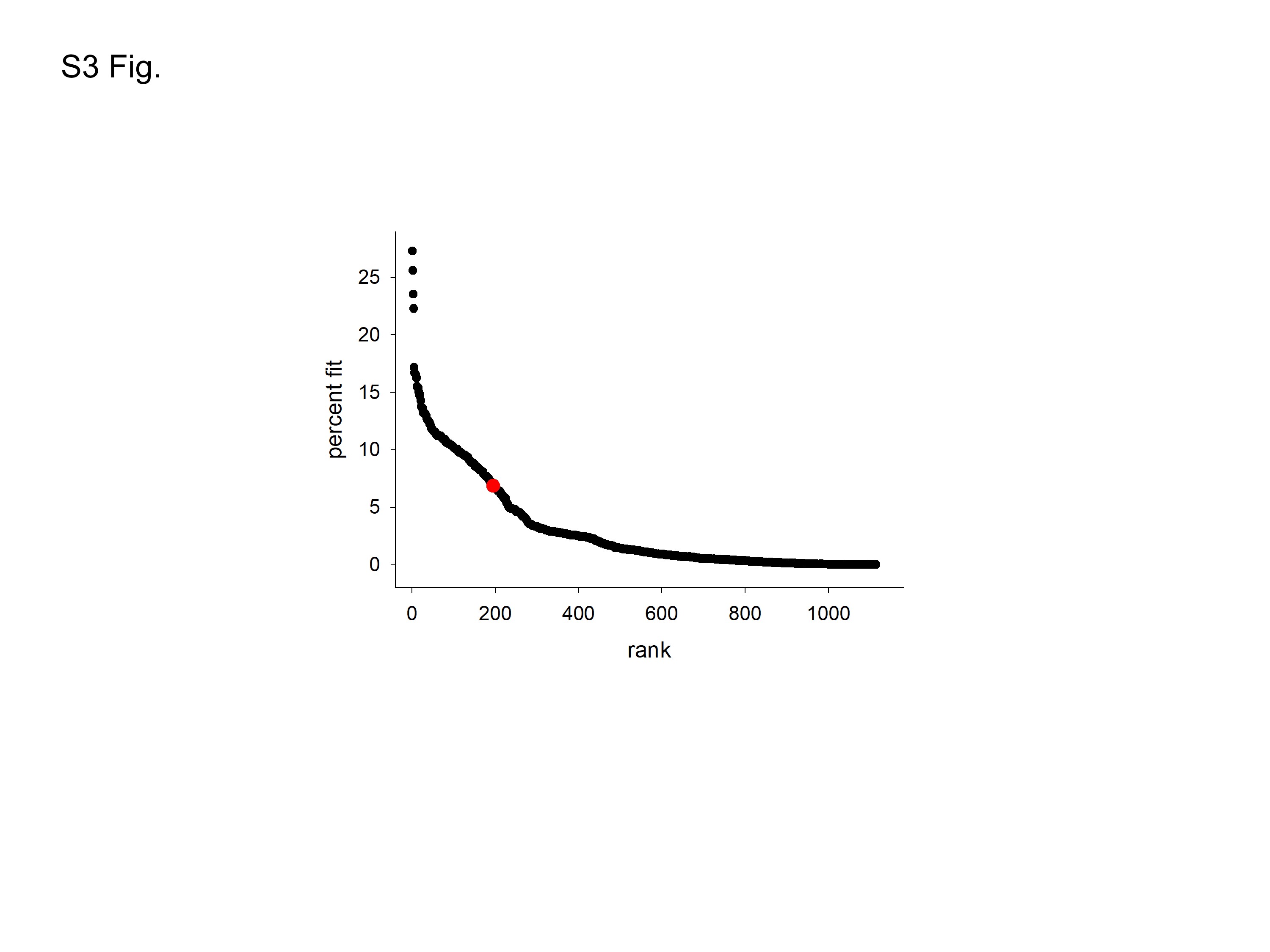

Supplement: S3 Fig — The Y axis shows the percent fit explained by RDA. Each dot represents an enzyme function as predicted from 16S sequences by PICRUSt2; tnaA is highlighted in red. The analysis is based on 24 pre-infection samples collected on days −5 and −3 and 1119 predicted enzyme abundance values. (JPG) [file pone.0324042.s005.jpg]
